# Repetitive Peripheral Magnetic Stimulation (rPMS) in Subjects With Migraine—Setup Presentation and Effects on Skeletal Musculature

**DOI:** 10.3389/fneur.2019.00738

**Published:** 2019-07-16

**Authors:** Tabea Renner, Nico Sollmann, Florian Trepte-Freisleder, Lucia Albers, Nina M. Mathonia, Michaela V. Bonfert, Helene König, Birgit Klose, Sandro M. Krieg, Florian Heinen, Lucia Gerstl, Mirjam N. Landgraf

**Affiliations:** ^1^Department of Pediatric Neurology and Developmental Medicine, LMU Center for Children With Medical Complexity, Dr. von Hauner Children's Hospital, LMU–University Hospital, Ludwig-Maximilians-Universität, Munich, Germany; ^2^Department of Diagnostic and Interventional Neuroradiology, Klinikum rechts der Isar, Technische Universität München, Munich, Germany; ^3^TUM-Neuroimaging Center, Klinikum rechts der Isar, Technische Universität München, Munich, Germany; ^4^Department of Neurosurgery, Klinikum rechts der Isar, Technische Universität München, Munich, Germany

**Keywords:** deltoid muscle, migraine, active myofascial trigger points, repetitive peripheral magnetic stimulation, trapezius muscle, trigemino-cervical complex

## Abstract

**Purpose:** Repetitive peripheral magnetic stimulation (rPMS) has been successfully applied recently in migraineurs to alleviate migraine symptoms. Symptom relief has been achieved by stimulating myofascial trigger points (mTrPs) of the trapezius muscles, which are considered part of the trigemino-cervical complex (TCC). However, effects on musculature have not been assessed in detail, and the specificity of effects to muscles considered part of the TCC yet has to be elucidated. Against this background, this study presents the setup of rPMS in migraine and evaluates effects on skeletal musculature.

**Materials and Methods:** Thirty-seven adults (mean age: 25.0 ± 4.1 years, 36 females) suffering from migraine and presenting mTrPs according to physical examination underwent rPMS either to mTrPs in the trapezius muscles (considered part of the TCC; *n* = 19) or deltoid muscles (considered not part of the TCC; *n* = 18) during six sessions over the course of 2 weeks. Standardized questionnaires were filled in to assess any adverse events and experience with rPMS as well as satisfaction and benefits from stimulation. Algometry was performed to evaluate changes in pressure pain thresholds (PPTs).

**Results:** All stimulation sessions were successfully performed without adverse events, with 84.2% of subjects of the trapezius group and 94.4% of subjects of the deltoid group describing rPMS as comfortable (*p* = 0.736). Muscular pain or tension improved in 73.7% of subjects of the trapezius group and in 61.1% of subjects of the deltoid group (*p* = 0.077). PPTs of the trapezius muscles clearly increased from the first to the last stimulation sessions—regardless of the stimulated muscle (rPMS to the trapezius or deltoid muscles). However, depending on the examined muscles the increase of PPTs differed significantly (subjects with stimulation of trapezius muscles: *p* = 0.021; subjects with stimulation of deltoid muscles: *p* = 0.080).

**Conclusion:** rPMS is a comfortable method in migraineurs that can improve local muscular pain or tension. Furthermore, it is able to increase directly and indirectly the PPTs of the trapezius muscles (considered part of the TCC) when applied over mTrPs, supporting the role of the TCC in migraineurs.

## Introduction

More than 1 billion people worldwide suffer from migraine according to a systematic analysis of the Global Burden of Disease Study of 2016 (1). Moreover, migraine has become the first cause of disability in subjects under 50 years of age (2). Although there has been a clear progress in knowledge in fields of epidemiology, etiology, acute and preventive treatment of migraine in the last decades, the distinct pathophysiology of migraine remains complex and multifactorial and is far from being entirely understood, with new aspects ranging from migraine-associated genes over specific neuropeptides to cervical afferences (3, 4).

Recent studies emphasize the association of neck pain with migraine, and there may also be a functional link between musculoskeletal dysfunction of the cranio-cervical region and migraine (5–10). Especially alterations in the trapezius muscles, semantically described as myofascial trigger points (mTrPs), seem to play a role, supporting the concept of a trigemino-cervical complex (TCC) that describes the convergence of cervical nociceptive sensory input of the radices C1-C3 with meningeal afferents in the caudal nuclei of the trigeminal nerve within the brainstem (11–17). Of note, studies have demonstrated a high occurrence of mTrPs in subjects with migraine and their associations with neck mobility (18–22).

Researchers try to modulate elements of the TCC by different invasive and non-invasive approaches in subjects with migraine to achieve symptom improvements. Neurosurgical, invasive occipital nerve stimulation (ONS) has been shown to modulate central pain processing mechanisms via inhibition of nociceptive input of cervical and meningeal afferents (23, 24). Non-invasive techniques are particularly attractive as they are well-tolerated, poor in side effects, and usually easy to apply (24, 25). Examples for centrally applied modalities are transcranial magnetic stimulation (TMS) (26–28) and transcranial direct current stimulation (tDCS) (29, 30). Non-invasive vagus nerve stimulation (VNS) (31–33) and supra-orbital nerve stimulation (SONS) (34, 35) represent further prominent interventions. Recently, repetitive peripheral magnetic stimulation (rPMS) has also been firstly applied in subjects with migraine, showing that the technique is applicable on the trapezius muscles and may successfully alleviate migraine symptoms (36). Furthermore, potential local effects of rPMS on the stimulated muscles by means of examining the pressure pain threshold (PPT) were analyzed, showing that the PPT in the trapezius muscles significantly increased during the course of six stimulation sessions, which supports the idea that rPMS has a positive, pain-reducing effect on the stimulated muscle in addition to its global effects on migraine frequency (36).

Previous studies were able to demonstrate that the PPT, which is defined as the cut-off between mere pressure and pressure-induced painful perception, tends to be decreased in the cranio-cervical region among subjects with migraine (7, 37–40). This supports the importance of muscular alterations and cranio-cervical hyperalgesia in headache disorders and provides further evidence that there might be a close link between peripheral sensitization and central nociception (7, 36, 40). However, although there is a considerable body of literature analyzing the PPT of different muscles of the cranio-cervical region in patients with migraine (e.g., trapezius, sternocleidomastoid, splenius, levator scapulae, or scalene muscles), none of the studies is examining in detail the larger shoulder girdle by comparing its muscles being involved in the TCC with those not being supposed to be part of the TCC (41, 42). Furthermore, there is a lack of evidence regarding the potential changes in PPTs in the course of modulation by techniques like rPMS considering muscles of the cranio-cervical region in comparison to muscles outside of the TCC. A potential specific effect of rPMS on muscles involved in the TCC, but not on those outside of the TCC, might further support the role of the TCC in migraine and the role of techniques like rPMS as valuable new modulatory approaches.

Against this background, the present study aims on demonstrating and evaluating the feasibility of rPMS delivered to the trapezius muscles as structures belonging to the TCC and the deltoid muscles as structures outside of the concept of the TCC among subjects with migraine. Moreover, it specifically evaluates the effects of rPMS on musculature by means of measuring the PPTs by algometry at several time points in the course of rPMS application.

## Materials and Methods

### Ethics

The study was approved by the institutional review boards of both universities of Munich (TUM and LMU) and was conducted in accordance with the Declaration of Helsinki. Written informed consent was a precondition for study enrollment.

### Participants and Experimental Protocol

Participants were recruited by announcements in the hospitals and local libraries of the two universities of Munich. The announcements informed about inclusion and exclusion criteria as well as the study plan and potential side effects of rPMS.

Inclusion criteria were (1) age between 18 and 35 years, (2) migraine (according to the German version of the headache questionnaire modified according to the International Classification of Headache Disorders [ICHD], 3rd edition and its beta version (43–45)), (3) a frequency of 15–44 days of headache during the 90 days prior to the first rPMS session (according to the headache diary of the German Migraine and Headache Society [DMKG]), (4) at least one active mTrP in one of the upper trapezius muscles (according to manual palpation by a specialized physiotherapist), (5) no metallic implants (e.g., cochlear implants), and (6) written informed consent. Exclusion criteria were (1) any neurological diseases except for migraine, (2) intake of any medication for migraine prophylaxis, (3) any changes in hormonal contraception during or shortly prior to study participation, and (4) pregnancy.

Overall, 199 subjects were screened, with 37 subjects fulfilling the inclusion criteria. Participants were randomized into two groups (randomization ratio: 1:1; participants were randomized by drawing sheets of paper with the participants' names to assign them to one or the other group) to receive rPMS either on the trapezius muscles (trapezius group; *n* = 19) or the deltoid muscles (deltoid group; *n* = 18). Overall, six sessions of rPMS were conducted per subject during 2 consecutive weeks in regular intervals (e.g., Monday/Wednesday/Friday or Tuesday/Thursday/Saturday).

### Evaluation of Migraine and Questionnaires

For this study we applied the German version of the headache questionnaire modified according to the ICHD (3rd edition and its beta version) (43–45), the headache diary of the DMKG, the Migraine Disability Assessment (MIDAS) (46, 47), a self-designed questionnaire to evaluate adverse events and experience with rPMS, and a self-designed questionnaire to evaluate the participants' satisfaction with rPMS as they were used in a previous pilot study (36).

To verify migraine diagnosis, the subjects had to initially fill in the German version of the headache questionnaire modified according to the ICHD (3rd edition and its beta version). Only those who fulfilled the criteria of migraine (migraine ± aura and/or ± tension-type headache [TTH]) were chosen for study participation. Subsequently, they were instructed to fill in the headache diary of the DMKG on a daily basis the 90 days before the period of stimulation sessions. This tool is interrogating subjects about trigger mechanisms, intensity, duration, quality, localization, concomitant symptoms, drug intake, and pain relief of each headache event. Additionally, participants were advised to evaluate the impairment in different aspects of daily life (e.g., productivity, household, social life) by headache events during the course of the 90 days prior to rPMS using the MIDAS questionnaire, which had to be completed on the first day of rPMS intervention. We used the results of the DMKG headache diary and the MIDAS questionnaire to compare the two groups (trapezius group and deltoid group) concerning their baseline characteristics regarding migraine before intervention.

Directly after each of the six individual stimulation sessions, a self-designed questionnaire assessed adverse events and experience with rPMS, covering pain perceived during stimulation (yes/no), paresthesia (yes/no, description of the uncommon sensation, assessment of the occurrence of the uncommon sensation in motion, rest, or constantly), muscle cramps (yes/no), and comfort during stimulation (yes/no/undecided). Ninety days after the intervention the participants evaluated the subjective benefit of rPMS and their satisfaction with stimulation retrospectively, using again a self-designed questionnaire assessing overall comfort (yes/no/undecided), willingness to repeat or recommend the stimulation (yes/no), and any improvements regarding the muscular situation (yes/no/undecided).

### Assessment of Myofascial Trigger Points

A certified physiotherapist specialized in mTrP palpations examined all participants within the week prior to the first scheduled rPMS session to identify two active mTrPs or, alternatively, one active and one latent mTrP in the trapezius muscles and latent mTrPs in the deltoid muscles bilaterally. To qualify as an active mTrP, palpated points had to meet the following standard criteria: (1) a taut band with a sensitive spot must be palpable, (2) its palpation must induce a referred pain at the typical localization of the subject's headache, (3) palpation of the sensitive spot must lead to a spontaneous evasive movement called “jump sign” (11, 48–50). In contrast, a latent mTrP does not show referred pain during palpation, but meets the criteria of (1) a taut band with local hypersensitivity, and (2) “jump sign” (51).

In total, we aimed to identify four points in each participant, one mTrP within the trapezius muscles bilaterally, of which at least one had to meet criteria of an active mTrP, and one latent mTrP within the deltoid muscles bilaterally. Participants showing only a unilateral active mTrP on one trapezius muscle were subsequently examined on the corresponding region of the contralateral trapezius muscle to identify a latent mTrP. In case that a subject presented more than one active or latent mTrP in one muscle, the physiotherapist chose the point that was most painful to intense palpation, with the other points not being further considered in the study.

The two mTrPs within the trapezius muscles and within the deltoid muscles were marked with a waterproof pen and documented by photos immediately after definition by the physiotherapist. Furthermore, we used a measuring tape to evaluate the distance of the mTrPs from the vertebral column, using the seventh cervical vertebra and the acromion as reference structures. The measurements were noted and further also documented by photos. Additionally, the physiotherapist documented the results meticulously in anatomical drawings of the neck and shoulder muscles. According to randomization, we stimulated either the two mTrPs of the trapezius muscles (trapezius group) or the two mTrPs of the deltoid muscles (deltoid group).

### Determination of Pressure Pain Thresholds

Measurements of PPTs were performed by algometry three times per mTrP during each of the six rPMS sessions (7, 52). Specifically, three consecutive PPT measurements were performed separately for the two mTrPs in the trapezius muscles and the two mTrPs of the deltoid muscles immediately before and after application of rPMS. In this context, the PPT as measured by algometry was defined as the cut-off between mere pressure and pressure-induced painful perception (7, 37–40).

**Figure 1:**
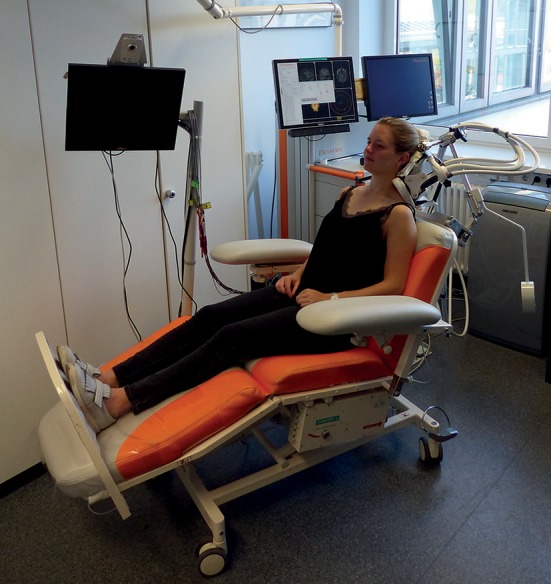
Setup of repetitive peripheral magnetic stimulation (rPMS). During algometry and rPMS, the subjects sat on a comfortable chair with armrests, headrest, and footplate in a relaxing position. Application of rPMS took place either to the myofascial trigger points (mTrPs) of the trapezius muscles (as shown in this case with the stimulation coil being placed on the left trapezius muscle with the help of a static coil holder) or to the mTrPs of the deltoid muscles depending on group assignment (trapezius group or deltoid group). The subjects were advised not to move during algometry or rPMS application and to rest in a relaxing position. Written informed consent was obtained from the subject of this figure to use this photo for publication.

**Figure 2:**
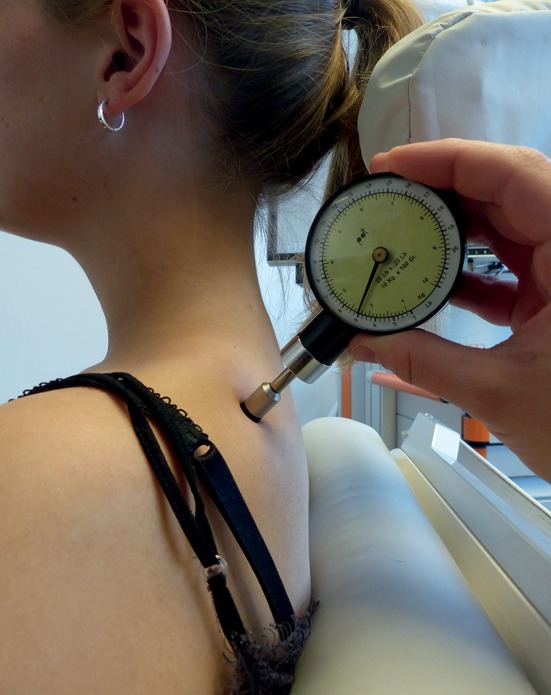
Measurements of the pressure pain threshold (PPT) by algometry. Measurements of PPTs were performed with a handheld algometer, which was placed perpendicularly to the skin with increasing pressure until the subject indicated that the local PPT was reached. Algometry was carried out on all four myofascial trigger points (mTrPs) in each subject. Specifically, three consecutive PPT measurements were performed separately for the two mTrPs in the trapezius muscles and the two mTrPs of the deltoid muscles prior and subsequent to the stimulation of each session.

During algometry and stimulation, the participants were seated on a comfortable chair with armrests, headrest, and footplate in a relaxing position in order to keep neck and shoulder muscles as less activated as possible (Figure 1). This position was kept for the initial and post-stimulation PPT measurements and during the entire application of rPMS. The investigator performed the algometry of the mTrPs on both sides by putting the algometer with a rubber tip of 1 cm^2^ perpendicularly to the skin whilst increasing the pressure slowly but steadily by 1 kg/s/cm^2^ until the participant indicated that the local PPT was reached (Figure 2).

We initiated the PPT measurements on the mTrP planned to be stimulated first during subsequent rPMS, followed by PPT measurements of the second ipsilateral mTrP. Subsequently, PPT measurements of the remaining two contralateral points were enchained. The same order of measurement was kept for post-stimulation PPT assessments. For both pre- and post-stimulation PPT measurements, there was a short break of 30 s to relax muscles again in between the three PPT measurements per point.

### Repetitive Peripheral Magnetic Stimulation

We used the Nexstim eXimia NBS System (version 4.3; Nexstim Plc. Helsinki, Finland) with a figure-of-eight stimulation coil for rPMS. This coil induces a focal field, combined with a cooling system to prevent overheating of the coil during pulse application. Depending on initial randomization, rPMS was applied either to the mTrPs of the trapezius muscles (trapezius group) or to the mTrPs of the deltoid muscles (deltoid group). Both sides were consecutively stimulated in each session, with the starting side being subject to randomization in the first session. During the following sessions the starting side was alternatingly chosen with respect to the first session per subject.

**Figure 3:**
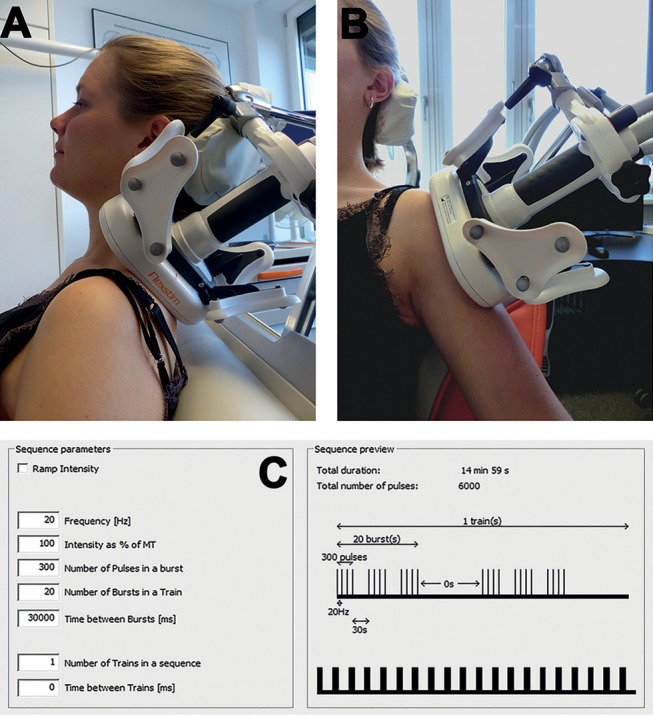
Stimulation by repetitive peripheral magnetic stimulation (rPMS). A figure-of-eight stimulation coil was used for rPMS, which was applied either to the mTrPs of the trapezius muscles (trapezius group) or to the mTrPs of the deltoid muscles (deltoid group) in the context of six stimulation sessions. Direct contact between the skin and the coil surface was ensured throughout, and the coil position was fixed by a static coil holder. In subjects of the trapezius group, the coil was centered and fixed above the previously identified mTrPs of the upper trapezius muscles perpendicularly to the anatomical course **(A)**. In subjects of the deltoid group, the coil was placed above the mTrPs of the deltoid muscles parallel to the anatomical course **(B)**. The stimulation protocol was the same in both groups (20 Hz) and took 15 min per side **(C)**. Written informed consent was obtained from the subject of this figure to use this photo for publication.

The stimulation coil was centered and fixed above the previously identified mTrPs of the upper trapezius muscles perpendicularly to the anatomical course or above the mTrPs of the lateral deltoid muscles parallel to the anatomical course with direct skin contact, depending on the group assignment (Figure 3). The coil was fixed by a coil holder to ensure a constant and stable position. During stimulation, the shoulder or upper arm elevated to a certain degree and sank down again during relaxation time (36). Skin contact as well as the position of the coil were ensured and regularly controlled during the whole stimulation and corrected, if necessary. The approach of coil positioning was the same for both sides and all points to be stimulated in each session and participant.

In total, six individual sessions were conducted within 2 consecutive weeks in each participant. Each session consisted of stimulation of the left and right mTrP of the trapezius muscles (trapezius group) or the left and right mTrP of the deltoid muscles (deltoid group), taking 15 min per side. For each side a total of 20 bursts consisting of 6,000 stimuli with a 20-Hz frequency were applied (Figure 3). A single burst consisted of 300 stimuli and lasted for 15 s, followed by a 30 s relaxation time (Figure 3). Furthermore, there was a break of a minimum of 2 min between rPMS to either side, used for changing the coil position for stimulation of the contralateral side.

Before the first stimulation session the intensity of rPMS was defined individually on the muscles to be stimulated according to assignment to the trapezius or deltoid group and was kept for the following sessions. Determination of the individual intensity was achieved in the previously described positions by starting stimulating with an intensity of 15% of the system's maximum output and increasing the intensity by steps of 5% while having the participants evaluating the comfort or discomfort/pain of each intensity on a visual analog scale (VAS) ranging from 0 to 10 (36). A score of 5 was defined as the cut-off value for painful sensation, i.e., we chose the intensity that was 5% lower than the intensity declared as 5 or higher on the VAS, thus regarded as discomfortable or painful, and used the corresponding intensity throughout for rPMS in the respective subject (36). The procedure of intensity determination was conducted on both sides for the muscles to be stimulated. In case that the results differed between sides, we chose the lower of the two intensities for stimulation of both sides.

### Data Analysis and Statistics

All statistical data analyses were performed using R software (version 3.1.0; The R Foundation for Statistical Computing, Vienna, Austria). A *p* < 0.05 was considered statistically significant.

For demographic data and headache characteristics (results of the DMKG headache calendar and MIDAS questionnaire), descriptive statistics including mean, standard deviation, median, and ranges or absolute and relative frequencies were calculated. To compare these data between subjects assigned to the trapezius or deltoid group, we used Wilcoxon-Mann-Whitney-U tests, Chi-squared, or Fisher tests. Results on experience with stimulation and adverse events and satisfaction with rPMS given as absolute and relative frequencies were compared between groups using Chi-squared or Fisher exact tests.

Regarding all analyses on PPTs as measured by algometry, we calculated the mean PPT out of the second and third measurement in each subject for each point separately (two mTrPs of the trapezius muscles and two mTrPs of the deltoid muscles), thus discarding the first measurements (53). First, for each session, differences between pre- and post-stimulation PPTs were assessed using Wilcoxon signed-rank tests, separately considering results among subjects stimulated on the trapezius or deltoid muscles and separately considering PPTs measured on the mTrPs in the right and left trapezius and deltoid muscles. Bonferroni correction for multiple testing was applied. Secondly, we compared the PPTs as measured initially before the first sessions to the corresponding values obtained after the last sessions, thus evaluating overall changes over the period of stimulations. To assess whether PPTs significantly increased, Wilcoxon signed-rank tests were used. As four tests per group were performed, Bonferroni correction for multiple testing was applied. Further, Friedman tests were used to assess whether increases in PPTs differed between examined muscles (right and left trapezius and deltoid muscles) in each group. For pairwise comparison between PPT increases in the examined muscles Nemenyi *post-hoc* tests were performed.

## Results

### Demographics and Baseline Characteristics

Table 1 shows demographics and baseline characteristics of the included subjects. We enrolled 37 young adults with an average age of 25.0 ± 4.1 years (range: 19–35 years), being randomly assigned to the trapezius group (*n* = 19) or the deltoid group (*n* = 18). Thirty-six of them were female, one was male. There were no significant differences between subjects receiving rPMS to the trapezius muscles and subjects receiving rPMS to the deltoid muscles regarding demographics or items of the headache diary of the DMKG or the MIDAS questionnaire (*p* > 0.05).

**Table 1:** Demographics and headache characteristics.

		**Trapezius group *N* = 19**	**Deltoid group *N* = 18**	***p***
		**Median (range) or % (*****N*****)**		
**SUBJECT CHARACTERISTICS**				
Age (in years)^a^		25.0 (19–35)	24.5 (19–32)	0.702
Female sex^b^		100.0 (19)	94.4 (17)	0.978
Type of migraine^c^	Migraine without aura	47.4 (9)	27.8 (5)	0.229
	Migraine with aura	36.8 (7)	16.7 (3)	
	Migraine without aura and TTH	10.5 (2)	27.8 (5)	
	Migraine with aura and TTH	5.2 (1)	27.8 (5)	
**HEADACHE DIARY OF THE DMKG (DAILY OVER THE COURSE OF 90 DAYS PRIOR TO INTERVENTION)**				
Number of days with headache^a^		23 (17–37)	20 (15–40)	0.057
Cumulative duration (hours)^a^		194 (78–429)	121 (60–482)	0.448
Average intensity (according to VAS)^a^		5.3 (3.5–6.9)	5.2 (3.9–6.5)	0.727
**MIDAS QUESTIONNAIRE (FOR THE 90 DAYS BEFORE INTERVENTION)**				
Missing school/work (days)^a^		1 (0–5)	1 (0–12)	0.405
Productivity at school/work reduced by half (days)^a^		10 (2–20)	7.5 (3–23)	0.247
Could not do household work (days)^a^		5 (0–11)	4.5 (0–18)	0.903
Household work productivity reduced by half (days)^a^		5 (0–15)	6 (0–14)	0.843
Missing family, social, or leisure activities (days)^a^		3 (0–10)	4.5 (0–17)	0.375

*This table shows cohort characteristics including details on headache (migraine with/without aura and with/without tension-type headache [TTH]) according to the headache diary of the German Migraine and Headache Society (DMKG) and the Migraine Disability Assessment (MIDAS) questionnaire. Average of headache intensity was measured with the help of a visual analog scale (VAS)*.

a *Wilcoxon-Mann-Whitney-U test*.

b *Chi-squared test*.

c *Fisher test*.

All participants presented with high-frequency episodic migraine and had one latent mTrP in each of the deltoid muscles. Moreover, all enrolled subjects showed at least one active mTrP in one of the trapezius muscles. In case that only a unilateral active mTrP was found in one of the trapezius muscles, a latent mTrP was identified on the contralateral side.

### Feasibility of rPMS and Adverse Events

Six single sessions of rPMS to either the mTrPs of the trapezius muscles or mTrPs of the deltoid muscles were feasible in all participants. There were no dropouts during the 2 weeks of application of rPMS.

Table 2 provides a summary of the evaluation of rPMS effects for all sessions stratified by group. During the 222 conducted stimulation sessions (114 stimulation sessions in the trapezius group and 108 stimulation sessions in the deltoid group), no adverse events occurred. According to the post-interventional assessment, high fractions of 81.6% of the conducted sessions among subjects of the trapezius group and 72.2% of the sessions among the subjects assigned to the deltoid group were described as comfortable (*p* = 0.220). Overall, only 1.7% of sessions were experienced as painful according to evaluations in the trapezius group, with no sessions performed in the deltoid group being declared as painful (*p* = 0.498). Uncommon sensations in the stimulated area, evaluated in terms of sensory function, were overall equally common in both groups (trapezius group: 28.1% of sessions, deltoid group: 26.9% of sessions; *p* = 0.958), with a significant difference regarding the feeling of numbness between groups (trapezius group: 3.1% of sessions, deltoid group: 20.7% of sessions; *p* = 0.046). Other evaluated parameters were again equally distributed between the sessions of both groups (*p* > 0.05).

**Table 2:** Experience with stimulation and adverse events.

		**Trapezius group *N* = 19**	**Deltoid group *N* = 18**	***p***
		**% (*****N*****)**		
Did you perceive the stimulation as painful?	Yes	1.7 (2)	0.0 (0)	0.498
Do you feel an uncommon sensation in the stimulated area?	Yes	28.1 (32)	26.9 (29)	0.958
What were the characteristics of the uncommon sensation in the stimulated area?	Tingling	40.6 (13)	48.3 (14)	0.732
	Muscle ache	18.8 (6)	37.9 (11)	0.167
	Numbness	3.1 (1)	20.7 (6)	***0.046***
	Cold/warmth	43.8 (14)	17.2 (5)	0.050
	Burning sensation	3.1 (1)	0.0 (0)	1
	Furry feeling	6.3 (2)	0.0 (0)	0.493
	Post vaccination	0.0 (0)	3.5 (1)	0.475
	Pressure	15.6 (5)	0.0 (0)	0.054
If yes, does the sensation occur in motion, in rest or constantly?	In motion	13.8 (4)	33.3 (3)	0.405
	In rest	31.0 (9)	33.3 (3)	
	Constantly	55.2 (16)	33.3 (3)	
Did any muscular cramps occur during stimulation?	Yes	0.0 (0)	0.0 (0)	1
Has the treatment been comfortable?	No	9.6 (11)	16.7 (18)	0.220
	Yes	81.6 (93)	72.2 (78)	
	Undecided	8.8 (10)	11.1 (12)	

*This table shows the results of a self-designed questionnaire to evaluate adverse events and experience with stimulation, which was assessed directly after each of the six individual stimulation sessions per subject*.

*Chi-squared test or Fisher test (for rare events with <5 observations for one of the tested groups; statistically significant p-values are printed in bold)*.

In a single subject of the deltoid group (female, 30 years), there was a dysesthesia occurring 48 h after the fourth stimulation session. The dysesthesia was reported to have started on the right arm, subsequently spreading to the left arm. Improvement of symptoms was achieved with intake of nonsteroidal analgesic drugs after 24 h, with symptoms disappearing 72 h after onset. No residuum was left. The subject described the dysesthesia to be similar, but slightly more prominent than her well-known sensations during migraine attacks. The participant decided to continue with the remaining rPMS sessions.

### Pressure Pain Thresholds

Table 3 presents the PPTs of the examined muscles of both groups in the course of the six stimulation sessions. Concerning the first and second session, the PPTs did not significantly change in any of the measured muscles when considering measurements in the trapezius and deltoid group. From the third session on, significantly higher PPTs were observed when comparing pre- to post-interventional algometry for several of the points in both groups.

**Table 3:** Evaluation of pressure pain thresholds (PPTs) by algometry—Part I.

**Session**		**1**		**2**		**3**		**4**		**5**		**6**	
		**Median (range)**	***p***	**Median (range)**	***p***	**Median (range)**	***p***	**Median (range)**	***p***	**Median (range)**	***p***	**Median (range)**	***p***
**STIMULATION OF TRAPEZIUS MUSCLE**													
Trapezius muscle—right side	Pre	2.1 (0.9–3.4)	0.352	1.7 (0.6–3.6)	*0.019*	1.8 (0.8–4.6)	*0.008*	1.9 (0.7–4.3)	*0.003*	1.8 (0.7–4.4)	*0.033*	2.0 (0.7–3.8)	*0.003*
	Post	2.0 (1–3.4)		1.8 (1.0–3.7)		1.9 (0.9–5.3)		2.1 (0.8–5.6)		2.0 (0.8–5.7)		2.5 (0.6–5.1)	
Trapezius muscle—left side	Pre	1.4 (1.0–3.4)	0.103	1.4 (0.6–3.6)	0.171	1.6 (0.6–4.2)	*0.039*	1.6 (0.7–3.7)	***0.002***	1.8 (0.7–3.6)	*0.018*	2.0 (0.7–5.8)	*0.010*
	Post	1.8 (0.9–2.6)		1.7 (0.7–3.7)		1.8 (0.6–4.6)		1.9 (0.8–4.6)		2.0 (0.8–4.0)		2.5 (0.9–5.2)	
Deltoid muscle—right side	Pre	1.4 (0.6–2.3)	0.472	1.4 (0.6–2.2)	*0.041*	1.4 (0.6–2.3)	*0.014*	1.3 (0.7–3.3)	0.184	1.3 (0.6–2.2)	*0.039*	1.2 (0.5–2.2)	*0.016*
	Post	1.4 (0.8–2.5)		1.3 (0.8–2.2)		1.5 (0.7–2.5)		1.3 (0.6–3.5)		1.5 (0.6–2.9)		1.3 (0.6–2.5)	
Deltoid muscle—left side	Pre	1.3 (0.7–2.1)	0.235	1.3 (0.6–2.3)	0.258	1.2 (0.6–2.2)	***0.001***	1.3 (0.6–2.5)	0.117	1.2 (0.7–2.2)	*0.032*	1.2 (0.6–2.4)	*0.028*
	Post	1.3 (0.7–2.4)		1.2 (0.7–2.6)		1.4 (0.6–2.6)		1.4 (0.6–2.4)		1.4 (0.7–2.0)		1.4 (0.6–2.7)	
**STIMULATION OF DELTOID MUSCLE**													
Trapezius muscle—right side	Pre	1.4 (0.8–5.7)	0.053	1.8 (0.8–6.2)	*0.008*	2.1 (0.8–6.6)	0.107	1.7 (0.7–5.4)	***0.0001***	2.0 (0.7–7.2)	***0.002***	1.8 (0.6–5.8)	***0.001***
	Post	1.9 (0.9–6.7)		2.2 (0.8–8.2)		2.2 (0.8–8.8)		2.5 (0.7–6.4)		2.5 (0.6–8.4)		2.2 (0.8–6.8)	
Trapezius muscle—left side	Pre	1.9 (0.7–4.5)	0.065	1.9 (0.6–5.7)	*0.012*	1.9 (0.6–6.5)	*0.004*	2.1 (0.8–6.3)	0.850	1.9 (1.0–6.4)	***0.001***	2.1 (0.8–7.1)	*0.012*
	Post	2.0 (0.7–5.3)		2.0 (0.8–10.1)		2.2 (0.8–7.7)		2.2 (0.8–5.8)		2.2 (1.1–7.0)		2.3 (1.1–6.0)	
Deltoid muscle—right side	Pre	1.6 (0.7–2.7)	0.061	1.4 (0.7–3.0)	*0.006*	1.5 (0.8–3.1)	*0.018*	1.4 (0.8–4.3)	*0.012*	1.3 (0.7–2.6)	***0.001***	1.3 (0.6–3.2)	*0.003*
	Post	1.7 (0.7–4.5)		1.5 (1.0–4.6)		1.6 (0.8–4.5)		1.6 (0.7–5.1)		1.6 (0.8–3.4)		1.5 (1.0–3.7)	
Deltoid muscle—left side	Pre	1.4 (0.7–3.0)	0.156	1.1 (0.6–3.5)	0.231	1.2 (0.8–3.5)	***0.002***	1.3 (0.7–2.8)	***<0.0001***	1.2 (0.7–2.6)	*0.029*	1.4 (0.4–2.7)	*0.011*
	Post	1.5 (0.6–3.3)		1.4 (0.6–2.9)		1.4 (0.8–3.9)		1.4 (0.8–3.7)		1.4 (0.8–2.9)		1.5 (0.7–2.9)	

*This table shows the results of algometry for each session, which was used to determine PPTs above the myofascial trigger points (mTrPs) of the trapezius and deltoid muscles. Three consecutive PPT measurements were performed separately for the two mTrPs in the trapezius muscles and for the two mTrPs of the deltoid muscles immediately before and after stimulation. The mean PPTs out of the second and third measurements were calculated in each subject for each point, thus discarding the first measurements*.

*Wilcoxon signed-rank test (with Bonferroni correction for multiple testing; statistically significant p-values after correction for multiple testing are printed in bold, statistically significant p-values that did not survive correction for multiple testing are printed in italics)*.

Table 4 compares the first measurement of the PPT before the first stimulation with the last measured PPT after the sixth stimulation session. When measuring the PPT of the trapezius muscles, there was an increase from the first to the last measurement regardless of the stimulated muscle, i.e., increased PPT values were observed in subjects stimulated on the deltoid muscles and in subjects stimulated on the trapezius muscles (by a median value between 0.4 and 0.7, respectively). However, significantly elevated values that survived correction for multiple comparisons were found only in the left trapezius muscles (subjects with stimulation of trapezius muscles: *p* = 0.005; subjects with stimulation of deltoid muscles: *p* = 0.009). In contrast, PPTs of the deltoid muscles did not significantly change when comparing the first to the last measurements with median increases between 0.1 and 0.3, respectively. The Friedman test confirmed that depending on the examined muscles the increase of PPTs differed significantly (subjects with stimulation of trapezius muscles: *p* = 0.021; subjects with stimulation of deltoid muscles: *p* = 0.080). Pairwise comparison resulted in significantly higher PPT increases in the left trapezius muscle compared to the right deltoid muscle in subjects with stimulation of the trapezius muscles and in significantly higher PPT increases in the left trapezius muscle compared to both deltoid muscles in subjects with stimulation of the deltoid muscles.

**Table 4:** Evaluation of pressure pain thresholds (PPTs) by algometry—Part II.

	**Trapezius muscle—right side**	**Trapezius muscle—left side**	**Deltoid muscle—right side**	**Deltoid muscle—left side**
**STIMULATION OF TRAPEZIUS MUSCLE**				
PPT pre first session median (range)	2.1 (0.9–3.4)	1.4 (1.0–3.4)	1.4 (0.6–2.3)	1.3 (0.7–2.1)
PPT post sixth session median (range)	2.5 (0.6–5.1)	2.5 (0.9–5.2)	1.3 (0.6–2.5)	1.4 (0.6–2.7)
*P*-value for comparison between first and sixth session^a^	0.080	***0.005***	0.167	*0.019*
Difference between PPTs post sixth and pre first session median (range)	0.4 (−1.1–2.5)	0.6 (−0.5–2.6)	0.1 (−1.1–1.5)	0.2 (−0.5–1.0)
*P*-value for comparison of PPT differences between examined muscles^b^	***0.021***			
*P*-values for pairwise comparison of PPT differences between examined muscles^c^	- Trapezius muscle left side and deltoid muscle right side: *p* = 0.017 - There were no significant differences between any other pairs			
**STIMULATION OF DELTOID MUSCLE**				
PPT pre first session median (range)	1.4 (0.8–5.7)	1.9 (0.7–4.5)	1.6 (0.7–2.7)	1.4 (0.7–3.0)
PPT post sixth session median (range)	2.2 (0.8–6.8)	2.3 (1.1–6.0)	1.5 (1.0–3.7)	1.5 (0.7–2.9)
*P*-value for comparison between first and sixth session^a^	*0.017*	***0.009***	0.327	0.486
Difference between PPTs post sixth and pre first session median (range)	0.7 (−1.1–3.1)	0.7 (−0.8–1)	0.3 (−0.8–1)	0.2 (−1.5–1.4)
*P*-value for comparison of PPT differences between examined muscles^b^	0.080			
*P*-values for pairwise comparison of PPT differences between examined muscles^c^	- Trapezius muscle left side and deltoid muscle right side: *p* = 0.04 - Trapezius muscle left side and deltoid muscle left side: *p* = 0.03 - There were no significant differences between any other pairs			

*This table shows the results of algometry of the initial measurement prior to the first stimulation session and the last measurement subsequent to the last stimulation session. The mean PPTs out of the second and third measurements were calculated in each subject for each point, thus discarding the first measurements*.

a *Wilcoxon singed-rank test (with Bonferroni correction for multiple testing; statistically significant p-values after correction for multiple testing are printed in bold, statistically significant p-values that did not survive correction for multiple testing are printed in italics)*.

b *Friedman test*.

c *Nemenyi post-hoc test*.

### Participant Satisfaction With rPMS

Table 5 gives an overview of the participants' subjective satisfaction with rPMS as evaluated 90 days after the last rPMS session. The majority of both groups retrospectively indicated rPMS to be comfortable (trapezius group: 84.2% of subjects, deltoid group: 94.4% of subjects; *p* = 0.736). More importantly, muscular pain or tension was reported to be improved in considerable fractions of 73.7% of subjects of the trapezius group and 61.1% of subjects of the deltoid group, yet with a statistical trend between groups (*p* = 0.077).

**Table 5:** Satisfaction with stimulation.

		**Trapezius group *N* = 19**	**Deltoid group *N* = 18**	***p***
		**% (*****N*****)**		
Has the stimulation been comfortable?	No	10.5 (2)	0.0 (0)	0.736
	Yes	84.2 (16)	94.4 (17)	
	Undecided	5.3 (1)	5.6 (1)	
Would you repeat the stimulation?	No	5.3 (1)	11.1 (2)	0.604
Would you recommend the stimulation for migraine?	No	10.5 (2)	16.7 (3)	0.660
	Yes	89.5 (17)	83.3 (15)	
Did the stimulation improve the muscular situation?	No	5.3 (1)	33.3 (6)	
	Yes	73.7 (14)	61.1 (11)	0.077
	Undecided	21.1 (4)	5.6 (4)	

*This table shows the results of a self-designed questionnaire to evaluate the subjective benefit from stimulation, which was assessed 90 days after the last stimulation session in each subject*.

*Fisher test*.

## Discussion

This study evaluated the feasibility and effects of rPMS delivered to the trapezius muscles, which are considered as structures belonging to the TCC, and the deltoid muscles as structures not being part of the TCC among subjects suffering from high-frequency episodic migraine. Regarding feasibility, all stimulation sessions were successfully performed without dropouts, technical problems, or lasting adverse events, and the majority of sessions was described as comfortable among subjects of both groups according to immediate post-interventional assessments as well as evaluations 90 days after the last rPMS session (Tables 2, 5). Concerning local effects within the muscles tested, PPTs as measured by algometry increased within the context of a single stimulation session when considering the third and later sessions (Table 3). More importantly, we found increases in the PPTs of the trapezius muscles from the first to the last measurements—regardless of the stimulated muscle (Table 4). Furthermore, depending on the examined muscles the increase of PPTs differed, with subjects stimulated on the trapezius muscles showing significant PPT differences (Table 4).

Various non-invasive techniques have been applied in subjects with migraine with the intention to alleviate symptoms via neuromodulation (24, 25). In this context, centrally applied modalities such as TMS (26–28) and tDCS (29, 30) as well as peripheral approaches such as VNS (31–33) and SONS (34, 35) are among the most common options. A new non-invasive technique in the field of migraine is represented by rPMS, which is an especially attractive alternative to these methods as it could induce both focal and central effects simultaneously when applied over muscles of the neck and shoulder area. On the one hand, rPMS can have influence on muscular structures, e.g. by increasing PPTs, and thus can be able to alleviate conditions like myofascial pain, neuropathic pain, or chronic pain (36, 52, 54–57). On the other hand, it was shown that rPMS—although applied peripherally—has central effects as well and is able to influence neuroplasticity, probably by increased proprioceptive inflow (58). Especially in migraine, muscular tenderness and hyperalgesia in neck and shoulder muscles are known for being linked to the incidence of migraine and the occurrence of its attacks (6, 8, 10, 59, 60). This interaction may be related to the nociceptive input of the radices C1-C3, which innervate the neck muscles and are converging with meningeal afferents in the caudal nuclei of the trigeminal nerve in the brainstem (13, 17). Central convergence and peripheral sensitization of trigemino-cervical neurons are the main aspects of the concept of the TCC, which aims to explain the complex pathogenesis of migraine associated with neck pain (13). Of note, investigations were indeed successful in triggering headache by manual palpation of mTrPs in the neck and shoulder region (19, 61). Hence, since rPMS seems to be able to approach both central and peripheral components of the TCC—as ONS is suggested to do as well—it might represent a promising and novel technique for effective interventions in subjects with migraine. Advantages over ONS are based on the non-invasive nature, ease of application, low rates of complications, and cost efficiency of the method. Importantly, the implementation of rPMS into treatment protocols is not subjected to refractory migraine; instead, like other neuromodulation approaches, it could be applied in different types or stages of migraine (24, 62).

To date, rPMS has been applied to active mTrPs of the trapezius muscles in subjects with migraine in one pilot study (36). This small study enrolled 20 young, predominantly female adults suffering from migraine, conducted six rPMS sessions, and evaluated acceptance and feasibility, performed algometry, and assessed potential impact on migraine (36). In both the present study as well as the previous pilot study using a similar setup and stimulation protocol, there were no dropouts or technical problems (36). Moreover, no lasting adverse events occurred during the entire study period, and single rPMS sessions were predominantly rated as comfortable (81.6% of the trapezius group and 72.2% of the deltoid group, Table 2). These rates are even higher than in the previous pilot trial on rPMS in migraine where rPMS was rated as pleasant regarding 55.8% of the sessions (36). Moreover, a high acceptance rate (94.7% of the trapezius group and 88.9% of the deltoid group, Table 5) as well as a high rate of recommendation of rPMS (89.5% of the trapezius group and 83.3% of the deltoid group, Table 5) were observed among participants without significant differences between the trapezius and deltoid group. Similarly, the previous pilot study reported on 100.0% of the participants willing to repeat rPMS while 90.0% would recommend it (36). Thus, rPMS appears a safe and well tolerable non-invasive technique that shows high acceptance among migraineurs who underwent stimulations, which seems a cornerstone for compliance and potential future transfer into clinics.

Previous research has shown that pressure pain sensitivity in the cranio-cervical region is generally elevated in subjects with migraine when compared to healthy controls (37, 38, 40). Consequently, subjects with migraine suffer more often from neck pain and cranio-cervical hyperalgesia, which is linked to musculoskeletal dysfunction (6, 7, 9, 10). Such hyperalgesia can be detected by measuring PPTs, and corresponding to elevated pain sensitivity, PPTs are regularly lower in the cranio-cervical region of patients with migraine than in healthy controls (7, 37, 39, 41, 53). In the present study, rPMS was indeed able to lead to a change in PPTs during the course of single rPMS sessions (Table 3). Increases in PPTs over the course of single rPMS sessions and particularly over the course of a 2-weeks interval of stimulation, as observed in the present study, seem to reflect improvements in hyperalgesia in migraineurs as measured by algometry. The finding that we did not observe a clear increase in PPTs in the course of the first sessions might implicate that only one session might not be able to change local conditions of neck and shoulder muscles, but repeated, thus multiple rPMS sessions seem potent enough to increase PPTs. This seems in good accordance with the previous pilot study that has also reported on increases in PPTs in the course of six rPMS sessions, but did only use stimulation of the trapezius muscles (36).

Of note, the present study did not only find increases in PPTs when comparing measurements before and after stimulation for single sessions; instead, we also found increased PPTs for the trapezius muscles when comparing the PPTs before the first stimulation with the very last measurement after the sixth rPMS session—regardless of the muscle that had been stimulated (Table 4). The finding that depending on the examined muscles the increase of PPTs differed (subjects with stimulation of trapezius muscles showed significant results whereas subjects with stimulation of deltoid muscles did not) might be explained within the concept of the TCC. The TCC claims that peripheral sensitization and central convergence of nociceptive afferents of C1-C3 could explain migraine pathogenesis in relation to neck pain (13). We hypothesize that the trapezius muscle that is considered part of the TCC in migraine might be more prone to improvements in hyperalgesia following rPMS than other adjacent muscles. This might be the result of central modulations probably reflected by neuroplasticity and increased proprioceptive inflow, features that have actually been observed in the course of rPMS elsewhere (58). In contrast, the deltoid muscles might not profit in the same way from rPMS, even not when stimulated directly, which might be related to missing access to the loops of the TCC that might be restricted to structures like the trapezius muscles. Hence, within the concept of the TCC in subjects with migraine, the trapezius muscles seem to be capable of responding better to both indirect and direct stimulation effects. Furthermore, the fact that the PPTs of the trapezius muscles increased even with rPMS to the deltoid muscles could be explained by a co-functional elevation of the shoulder and, thus, passive movement of the trapezius muscles during stimulation. Other explanations might be that there are connections between the trapezius and the deltoid muscles or that the afferents of both muscles converge at some point on the way to the brainstem. Thus, via measurements of effects of rPMS by algometry, this study emphasizes the importance of the trapezius muscles in the complex of the TCC and might support the assumption that the deltoid muscles are not primarily involved in the TCC (13–16).

Although this study provides new insights into rPMS and its effects on skeletal musculature in subjects with migraine, certain limitations need to be highlighted. With regards to study inclusion, the comparatively low number of participants in each group represents a shortcoming, together with the predominant enrollment of females over males. Second, the participants' narrow age range, which was between 18 and 35 years, as well as the focus on subjects with high-frequency episodic migraine might represent shortcomings as results obtained in this study might not be generalized with respect to migraineurs in different ages or with different frequency characteristics of migraine. Third, the inclusion of individuals suffering from migraine and TTH as well as individuals suffering only from migraine can be considered as a limitation as there is no evidence available regarding the issue how rPMS would influence TTH only. However, there is a high prevalence of TTH among migraineurs, similar to the prevalence among non-migraineurs (63). This shows that migraineurs suffering also from other headache disorders represent an important proportion of the population and should also be considered as participants. Fourth, the present study did not evaluate effects of rPMS applied to the trapezius or deltoid muscles on characteristics of migraine. Potential alleviating effects on the number of migraine attacks and migraine intensity, amongst other factors, have been suggested by a previous pilot study (36); however, further evidence for the positive impact of rPMS on migraine characteristics is needed.

With regards to the study's setup and design, the lack of a control condition to assess potential placebo or setting effects on PPTs reflects a potential shortcoming. Such a control condition might have been established by sham stimulation of the trapezius or deltoid muscles. A sham coil, i.e. a coil with a plastic tube to avoid direct contact between skin and coil, could be utilized to prevent actual local stimulation. In this case the participant would still experience the device's typical noise and direct skin contact, but would not experience any muscular contractions (58, 64). Another option could be a reduction in stimulation parameters like intensity and frequency to reduce effects of rPMS so that participants could perceive a clearly less remarkable contraction of the stimulated muscles (58). However, sham-controlled studies using either of these options cannot be realized that easily in case of rPMS where a missing stimulation effect on musculature is evidently experienced by study participants (65). Second, the exact localization of stimulation was defined according to previous manual palpation performed to detect active or latent mTrPs. Manual palpation is considered the gold standard for the identification of mTrPs since decades (25, 66); however, novel techniques like qualitative magnetic resonance imaging (MRI) or quantitative MRI using T2 mapping might be capable of visualizing and determining mTrPs more objectively, thus paving the way for navigated rPMS interventions (11, 12, 67). Third, this study only involves a certain neck muscle, the trapezius muscle, as a structure being part of the TCC and only one muscle, the deltoid muscle, that is not supposed to be involved in the TCC. Particularly stimulation to other muscles outside of the concept of the TCC and more distant to musculature considered part of the TCC might provide further evidence for our suggestion that structures of the TCC might be more prone to improvements in hyperalgesia following rPMS in migraine. Future studies could make advantage of novel MRI-guided rPMS approaches and might consider control conditions and further muscle groups for stimulation in the context of more advanced study setups.

## Conclusion

This study applied rPMS to mTrPs of trapezius muscles (considered part of the TCC) and mTrPs of deltoid muscles (considered not part of the TCC) in migraineurs. The approach showed to be feasible and comfortable, with improvements in local muscular pain or tension being evident. Particularly the mTrPs of the trapezius muscles were responding to stimulation via application of rPMS, suggesting that the trapezius muscles might play a more complex role not only in muscular interaction but also in the concept of the TCC. Further studies are needed to explore in more detail structures in and outside of the TCC as well as modulating local and central effects of rPMS in subjects with migraine.

## Data Availability

The datasets generated for this study are available on request to the corresponding author.

## Ethics Statement

The study was approved by the institutional review boards of both universities of Munich (Technical University of Munich, TUM, and Ludwig-Maximilians-University, LMU) and was conducted in accordance with the Declaration of Helsinki. Written informed consent was a precondition for study enrollment.

## Author Contributions

TR and NS: data acquisition, data handling, data analysis including statistics, data interpretation, literature research, drafting of the manuscript, and read and approved final version. FT-F: data acquisition, data handling, literature research, and read and approved final version. LA: data handling, data analysis including statistics, and read and approved final version. NM and MB: data interpretation, literature research, and read and approved final version. HK and BK: definition of mTrPs, data acquisition, and read and approved final version. SK: data handling, literature research, and read and approved final version. FH, LG, and ML: data acquisition, data handling, data analysis, data interpretation, literature research, drafting of the manuscript, and read and approved final version.

### Conflict of Interest Statement

NS received honoraria from Nexstim Plc (Helsinki, Finland). SK is consultant for Nexstim Plc (Helsinki, Finland). The remaining authors declare that the research was conducted in the absence of any commercial or financial relationships that could be construed as a potential conflict of interest.

## Abbreviations

DMKG: German Migraine and Headache Society
ICHD: International Classification of Headache Disorders
MIDAS: Migraine Disability Assessment
MRI: Magnetic resonance imaging
mTrP: Myofascial trigger point
ONS: Occipital nerve stimulation
PPT: Pressure pain threshold
rPMS: Repetitive peripheral magnetic stimulation
SONS: Supra-orbital nerve stimulation
TCC: Trigemino-cervical complex
tDCS: Transcranial direct current stimulation
TMS: Transcranial magnetic stimulation
TTH: Tension-type headache
VAS: Visual analog scale
VNS: Vagus nerve stimulation.
